# Exploring Comprehension Strategies of Modular Process Models: A Combined Eye-Tracking and Concurrent Think-Aloud Study

**DOI:** 10.3390/brainsci14040303

**Published:** 2024-03-23

**Authors:** Julia Baß, Michael Winter, Rüdiger Pryss, Manfred Reichert

**Affiliations:** 1 Institute of Databases and Information Systems, Ulm University, 89081 Ulm, Germany; manfred.reichert@uni-ulm.de; 2 Institute of Clinical Epidemiology and Biometry, University of Würzburg, 97070 Würzburg, Germany; michael.winter@uni-wuerzburg.de (M.W.); ruediger.pryss@uni-wuerzburg.de (R.P.)

**Keywords:** process model, modularization, comprehensibility, eye-tracking, think-aloud, study

## Abstract

The study of complex process models often encounters challenges in terms of comprehensibility. This paper explores using modularization as a strategy to mitigate such challenges, notably the reduction in complexity. Previous research has delved into the comprehensibility of modularized process models, yet an unresolved question about the cognitive factors at play during their comprehension still needs to be answered. Addressing the latter, the paper presents findings from an innovative study combining eye-tracking and concurrent think-aloud techniques involving 25 participants. The study aimed to comprehend how individuals comprehend process models when presented in three different modular formats: flattened process models, models with grouped elements, and models with subprocesses. The results shed light on varying comprehension strategies employed by participants when navigating through these modularized process models. The paper concludes by suggesting avenues for future research guided by these insights.

## 1. Introduction

**Figure 1:**
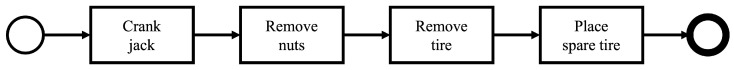
Process model (example tire change).

Process-oriented domains frequently encounter the challenge of managing highly complex process models. The complexity of these models arises from various factors, including the size of the process model, such as the number of activities and actors [1], and its structural composition, like element arrangement [2]. The size of a process model reflects the range and depth of issues it represents, where specific elements (e.g., activities, events) are selected and interconnected to depict these issues, as illustrated in Figure 1. Consequently, incorporating more issues leads to an increase in both the number of elements and the overall complexity of the model. Yet, this escalation in complexity is not without its pitfalls. Complex process models are notably more prone to errors [3]. They tend to include more formal errors, such as deadlocks [4]. A detailed examination of 2003 process models focused on error rates revealed that models with over 48 elements exhibit error-proneness exceeding 50% [4]. Supporting this finding, guidelines have been proposed recommending the subdivision of process models with more than 50 elements to mitigate error risks [5]. Alongside error-proneness, these complex models also impact comprehensibility [6]. To manage these complexities and associated challenges, process model modularization has been introduced as a solution [7]. This approach involves breaking down a monolithic process model into smaller, independent, and self-contained modules. Thus, modularization is a common approach to reduce the complexity of process models. Modularized process models are generally easier to comprehend, owing to their lower information density, which is a marked advantage over non-modularized models [7]. The efficacy of modularization has been demonstrated through its positive effects on comprehension, evidenced by increased accuracy in answering questions related to the process models [8]. However, implementing modularization requires careful consideration as it can vary in form and impact [9]. Different modularization techniques, such as creating subprocesses through a vertical hierarchy, can significantly influence the comprehensibility of the process models [10,11]. In conclusion, while modularization plays a critical role in ensuring the accuracy and representation of a process model, it can also have negative effects on comprehension. Therefore, comprehending how to effectively manage the modularized process model and comprehending how humans interpret these models is crucial. To the best of our knowledge, in the context of modularized process models, there is no work dealing with the interpretation of these models by focusing on comprehension strategies. Hence, the paper at hand covers this gap by presenting the results of a comprised eye-tracking and think-aloud study where participants had to search for activities in modularized process models.

This paper has the following structure: In Section 2, the related work is presented. Then, the theoretical backgrounds on modularization as well as process models are described in Section 3. Section 4 describes the materials and methods used in the conducted eye-tracking and think-aloud study. Moreover, in Section 5, the evaluated results of the study are presented and analyzed. In addition, limiting factors and implications for research are shown in this section. Finally, the conclusion and outlook on future work are presented in Section 6.

## 2. Related Work

Substantial research has been devoted to understanding how people comprehend process models, focusing on factors like size, notation, and color influencing comprehension outcomes, such as improved accuracy in answering related questions. For instance, research indicated that process models mapped in an upward direction could be more comprehensible compared to other orientations like straight or downward [12]. Next to factors like size, the modularization was an object of consideration. Ref. [10] compared different modularization types, including flattened process models, models with groups, and models with subprocesses, across various presentation mediums (e.g., paper, computer). Differences in comprehensibility and task performance were highlighted [10]. For instance, flattened process models have been shown to be more comprehensable, useful, and effective compared to models with subprocesses in a study involving large real-life process models. Next to the comparison of the mentioned modularization types, the modularization techniques horizontal, vertical, and orthogonal were considered in terms of process models [13,14]. Ref. [14] outlined that horizontal modularization appears to be more comprehensible than vertical and orthogonal modularization. This is in contrast to the results of [13,15], where horizontal modularization led to negative effects on comprehensibility. Therefore, so far, no common understanding has been reached regarding the impacts of the different modularization techniques on comprehensibility.

The concept of cognitive load has also been a focal point in this area of research. Studies suggest a reduced cognitive load can lead to better process comprehension [16]. Some aspects within process models have been identified as inherently more challenging to comprehend, suggesting a need for their modification or replacement [16]. Other research has examined the influence of process model modularization techniques (i.e., vertical, horizontal, orthogonal). Ref. [14] showed that in terms of the modeling element interactivity such as the presentation, the load is on a low up to medium level. But, when focusing on the mental effort, the load is medium up to high. This indicates that comprehending modularized process models is associated with exertions. Furthermore, ref. [15] outlines that horizontal modularization leads to a higher cognitive load than vertical modularization. Eye-tracking technology has been employed to gain further insights into how individuals comprehend process models. For example, a study used eye-tracking to observe how healthcare professionals comprehend process models, analyzing aspects like the number and duration of fixations, and concluded that comprehension often happens intuitively [17]. Another study focused on process model modularization techniques (i.e., vertical, horizontal, and orthogonal), and ref. [13] considered gaze patterns linked to reading and comprehension behavior. While comprehending modularized process models with a different level of complexity (i.e., low, medium, and high), six modularization-specific gaze patterns (e.g., orientation, comprehension) were identified in the eye-tracking study. Despite the prevalence of eye-tracking and cognitive load analysis methods, the think-aloud approach has been less frequently applied to process model comprehension. However, in a study examining user interaction with hybrid process artifacts, the retrospective think-aloud method (where participants report their thoughts after completing a task) was used alongside eye-tracking. This approach provided more profound insights into participant behavior and their challenges during task performance. For example, it was shown that DCR Graphs (declarative process models based on directed graphs) helped users gain an overview of the business process [18]. In other fields, such as usability, the think-aloud method is widely used due to its benefits in revealing cognitive processes [19,20]. However, each of these methodologies offers unique insights into the comprehension of process models. Therefore, appropriately selecting and combining these approaches is crucial for a comprehensive understanding of how individuals interact with and comprehend process models.

The integration of eye-tracking and think-aloud methodologies in studying process model comprehension, especially in modularized process models, has been relatively scarce. This paper presents a unique study exploring the cognitive aspects of searching for activities in modularized and non-modularized process models. Eye-tracking technology provides valuable insights into the comprehension process by capturing detailed information such as fixations and eye movements. Combined with the think-aloud method, this approach also uncovers the participants’ reasoning and motivations as they search for specific activities within the process models. The combination of eye-tracking data and participants’ verbalizations allows for identifying distinct comprehension strategies. These strategies illuminate the participants’ methods and approaches when navigating process model activities. This information is particularly valuable as it offers a window into the cognitive processes during interaction with process models. Consequently, the findings from this study lay the groundwork for developing targeted comprehension guidelines specifically tailored for modularized process models. By understanding how individuals engage with and interpret these models, the study contributes significantly to our knowledge of effective process model design and user interaction, highlighting the nuances of human cognition in the context of process model reading and comprehension.

## 3. Theoretical Background

**Figure 2:**
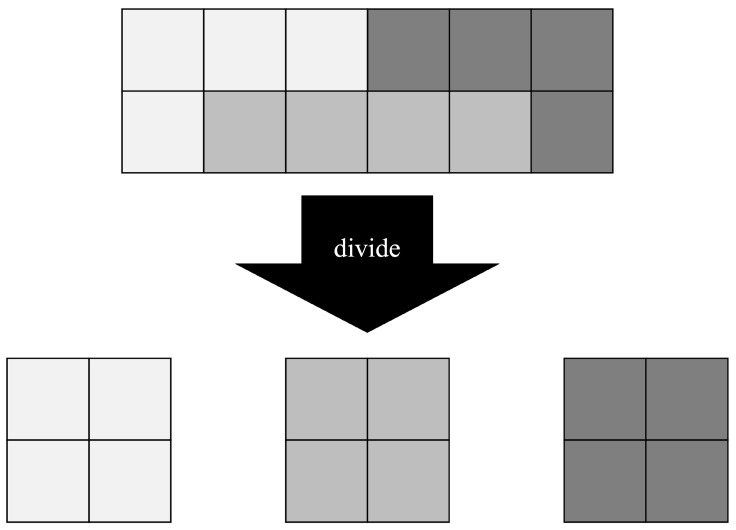
Modularization: from a monolith to single modules.

Modularization serves as an effective method for managing complexity within complex technological contexts [8]. It involves breaking down a monolithic structure into smaller, more manageable modules, as illustrated in Figure 2. These modules interact through specific interfaces, allowing for greater adaptability and independence. This adaptability enables individual modules to be easily exchanged or optimized [5]. Additionally, modularization facilitates the reuse of components, significantly enhancing efficiency [21].

**Figure 3:**
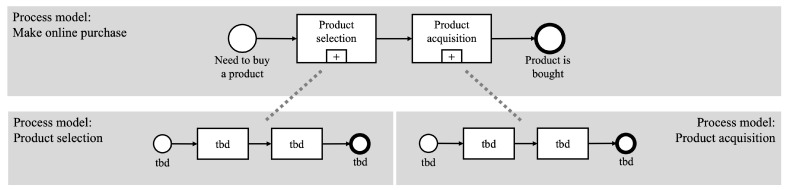
Example process model subdivision.

**Figure 4:**
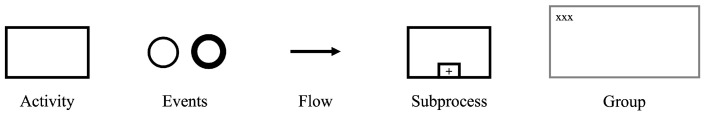
Modeling elements.

**Figure 5:**
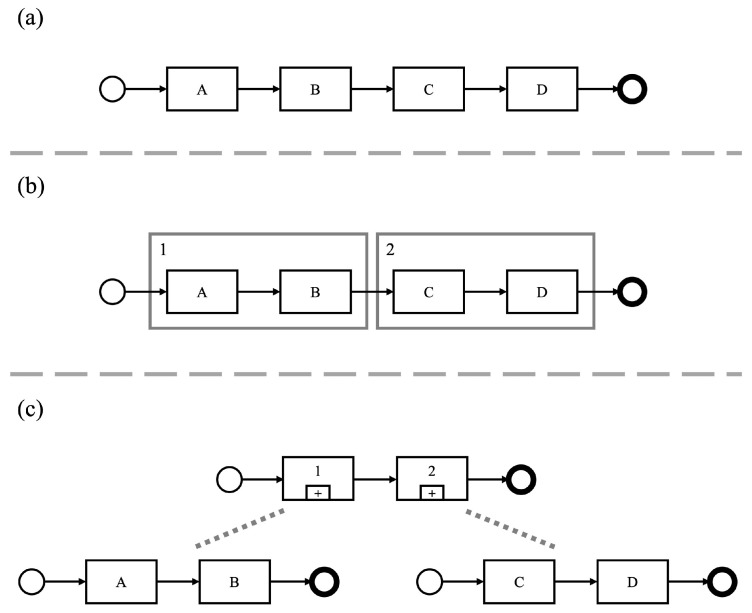
Modularized and non-modularized process models: (**a**) flattened process model, (**b**) modularization on the same level, and (**c**) modularization over different levels.

This concept has found widespread application across various domains, notably in software development, where it plays a critical role in system design and management [22,23]. In domains dealing with complex systems, such as aircraft systems, modularization creates a system hierarchy. Such a system hierarchy can be studied from [24], and it comprises several structural levels: the target system, subsystems, and system elements. The target system represents the highest level of the hierarchy, functioning as the overarching structural element or the root. It is further divided into multiple subsystems, or nodes, transforming what was once a singular system into a network of interconnected smaller systems [25]. These subsystems can be broken down even further into more detailed subdivisions. When a subsystem is not subdivided any further, it is identified as a system element, which represents the leaves of the hierarchy. Each system element engages in interactions with other elements [24]. Consequently, a system element is only partially autonomous. Modularization also plays a crucial role in the domain of process model comprehension. In this context, a more complex process model is divided into several smaller, more manageable models, enhancing reusability, flexibility, and complexity reduction [26]. For instance, consider the process model “make online purchase” depicted in Figure 3. This model, starting from the “need to buy a product” and culminating in the “product is bought”, can be segmented into smaller models or modules, such as “product selection” and “product acquisition”. To effectively represent the original process, these smaller models must be interconnected at their interfaces, for example, through linkages. Applying modularization to process models necessitates using additional elements, like subprocesses and linkages, which may vary depending on the process model notation. Figure 4 showcases various process model elements using the BPMN 2.0 notation for illustration purposes. BPMN 2.0 is a versatile notation allowing for the implementation of different types of modularization, as highlighted by [7]. This paper focuses exclusively on process models described using the BPMN 2.0 notation. The subsequent discussion will describe the elements used in the process models shown in Figure 5a–c, providing a detailed understanding of how these elements contribute to the overall structure and functionality of the modularized process models. The following elements are described:

- Activity: Atomic step in the process model.
- Event: Indicates that something has happened.
- Sequence Flow: Connects elements of the process model and indicates the direction.
- Subprocess: Abstraction of a section of the process, which is detailed in another process. As the information of the underlying process model is not directly visible and is hidden by the subprocess, the process models are on different levels.
- Group: Clusters several connected elements that belong together in terms of content.

In Figure 5, three different process model modularization types are visualized. These modularization types were also considered in [10,11]. By using these modularization types, refs. [10,11] showed the influence of grouping the content and subdividing the process across several levels on comprehensibility. In the following, different process model modularization types are introduced.

### 3.1. Flattened Process Models

In a flattened process model, modularization is not utilized. This is exemplified in Figure 5a, which displays a flattened process model. The model is characterized by a sequence of activities linked together by sequence flows. At the beginning of the process is a start event, marking the initiation of the workflow. Conversely, the process culminates in an end event, signifying its completion. This linear and straightforward structure allows for easy visualization and comprehension of the process flow from start to finish.

### 3.2. Modularization on the Same Level

In contrast to flattened process models, modularization at the same hierarchical level occurs when a process model is segmented without employing subprocesses. This type of modularization involves grouping elements, either by content or logic [27]. Various modularization techniques, such as vertical or horizontal approaches, can be used to group elements within process modeling. These different approaches, while aiming for a similar level of modularization, are executed using distinct elements.

In process models described in BPMN 2.0, modularization at the same level can be represented by elements like expanded subprocesses, link events, and groups. Figure 5b illustrates a process model that utilizes groups, an approach that extends beyond the structure of a flattened process model. In this model, a group is visually indicated by a frame encircling several elements, effectively clustering them together. The name or label of the group is typically positioned at the top of the group element, providing clear identification and contextual information about the grouped elements and their collective purpose within the overall process.

### 3.3. Modularization over Different Levels

Modularization across different levels involves structuring process models in a vertical hierarchy, distinguishing between high-level and detailed perspectives. High-level process models are characterized by abstract activities, which are essentially generic terms that offer an overview of the process but contain minimal detailed information. To enrich these abstract activities, or subprocesses, with necessary details, they are elaborated upon by more detailed, underlying process models.

Vertical modularization, which involves the use of subprocesses, effectively conceals intricate process details, making the overall model less overwhelming and easier to navigate [7,14,28,29]. This approach is particularly beneficial for enhancing the comprehensibility of complex and large process models [7]. Additionally, the application of subprocesses can reduce redundancy and promote reuse as a single subprocess can be referenced from multiple points within the model [7].

Figure 5c displays a process model that incorporates subprocesses. In this model, the upper layer represents the high-level process, including elements of the closed subprocess. These subprocess elements are linked to their respective underlying process models, which provide a more detailed and content-specific elaboration of the high-level abstract activities. This hierarchical arrangement facilitates a clearer comprehension of the process by allowing users to delve into details as needed, without overwhelming them with information at the initial, higher level of the process model.

## 4. Methods and Materials

### 4.1. Context Selection

Real-life process models often experience increased size and complexity when represented as single, monolithic structures [11]. Both the size of the model [1] and its complexity [6] significantly influence the level of difficulty of the process model comprehension. The modularization of process models has been shown to enhance comprehension, primarily because modules within these models encapsulate a more manageable amount of information [7]. Studies have documented the positive effects of process model modularization [8], yet there is also evidence suggesting that flattened process models, which lack subprocesses, are sometimes more straightforward and easier to comprehend.

To gain deeper insight into how different types of modularization affect the comprehension of process models, methodologies like eye-tracking and the think-aloud approach can be highly effective. Eye-tracking provides valuable data on where readers focus their attention and how they manage cognitive load while interacting with a process model. Concurrently, the think-aloud approach captures the thought processes and procedural steps that readers undertake during their engagement with the model. By combining these two methods, it becomes possible to identify and understand the various comprehension strategies employed by individuals when they interpret process models. This holistic approach allows for a more nuanced understanding of how different modularization techniques impact the readability and interpretability of process models.

In this study, which combines eye-tracking with concurrent think-aloud methodologies, we focus on the modularization types examined in [10]: flattened process models (➀), process models with groups (➁), and process models with subprocesses (➂). Our aim is to gain a deeper understanding of how readers comprehend these different types of process models. Drawing on the insights obtained through these selected methodologies, we have formulated the following four research questions (RQs) to guide our investigation:

- RQ1: Do different modularization types in process modeling influence the cognitive load when comprehending process models?
- RQ2: Do different modularization types in process modeling influence the comprehensibility when comprehending process models?
- RQ3: Do different modularization types in process modeling influence the performance when comprehending process models?
- RQ4: Which procedures are used when searching for activities?

### 4.2. Participants

A total of twenty-five participants were involved in the study: twenty participated in the study at Ulm University and five at the University of Würzburg. The participant group comprised twenty-two males and three females. Age-wise, two participants were under 25, twenty-one were between 25 and 35, and two were between 35 and 45 years old. Regarding educational background, nine participants held a Bachelor’s degree, fourteen had a Master’s degree, and two possessed a Ph.D. In terms of field of study, fourteen participants were from computer science, three from economics, two from economics and computer science, two from design, and four from other fields. Out of the total participants, 18 reported prior knowledge of process modeling. These participants were further required to complete a demographic questionnaire followed by 10 true-or-false questions to assess their comprehension of process models, particularly focusing on syntactical rules. On average, participants correctly answered 6.05 out of the 10 comprehension questions, as indicated by the mean score. The participant sample’s complete demographic and background details are presented in Table 1.

### 4.3. Materials

In this study, process models (the materials are available at: https://drive.google.com/drive/folders/1-nEPF2B5DY-tpwPF9Dt_hTNs8PLu-bwE?usp=sharing) were created. Thereby, the focus was on the respective modularization type (i.e., flattened process models ➀, process models with groups ➁, and process models with subprocesses ➂). The modularization type comprises modularized types such as non-modularized process models. The process models were expressed in terms of the BPMN 2.0 [30]. This modeling notation was selected for several reasons. BPMN 2.0 represents an ISO/EC standard [31] that is widely used (e.g., industry, research). Through BPMN 2.0, different scopes regarding processes can be covered (i.e., modeling, automation). In terms of process modeling, BPMN 2.0 includes a large range of modeling elements (e.g., gateways, events). Furthermore, BPMN 2.0 supports different approaches for modularization, such as collapsed or expanded subprocesses and groups [10]. In this study, the M = 75 BPMN 2.0 process model was created. As provided in Table 2, three process models were designed for the page type trial (i.e., one for each of the three modularization types). Hence, in total, 72 process models were created for the process scenarios (i.e., P1–P6). For each of the six process scenarios, a composition of the three modularization types such as four tasks was applied (i.e., 3 modularization types * 4 tasks = 12 process models per process scenario). Each of the four tasks involved finding an activity in the process model (e.g., “Find the activity ‘Buy flowers’”) by the participants. Further, the scenarios (e.g., refuel car) were kept simple to ensure that the participant can focus on the modularization types (i.e., ➀, ➁, ➂). Seven process model scenarios were modeled: flower purchase, refuel car, order food, prepare vacation, handling car breakdown, customer service coffee shop, and lending. Each process model was created in the three modularization types (i.e., ➀, ➁, ➂). For all three modularization types, the process models (i.e., P0–P6) consist of 13 activities on average. Next to activities, further common business process model elements such as events, roles, and flows were utilized in the process models (i.e., part of the process flow) to ensure a representation with a practical orientation. The different modularization types consist of the following average element numbers for P0–P6: 68 elements for ➀, 71 elements for ➁, and 89 elements for ➂. Table 2 provides details of the process models. According to the number of applied elements, the process models were suitable for modularization (i.e., more than 50 elements). Furthermore, a holistic visualization of the process models was realizable as the number of elements was not too high.

### 4.4. Performance Measures and Comprehension Strategies

In the study, the following performance has been evaluated (the materials are available at: https://drive.google.com/drive/folders/1DdlDWSra_pCSYnMybKLB6aidPZvnrgHH?usp=sharing):

- Cognitive load: Following the framework of Cognitive Load Theory [32], cognitive load encompasses three dimensions: intrinsic (ICL), germane (GCL), and extraneous (ECL) [33]. Intrinsic cognitive load refers to factors like prior knowledge and element interactivity. Germane cognitive load focuses on the cognitive resources necessary for learning and mental model construction. Extraneous cognitive load involves the presentation aspects of information, such as its complexity [34]. Cognitive load was a key focus of Research Question 1 (RQ1). To measure it, a standardized questionnaire from [35] with seven items (2 for ICL, 2 for GCL, and 3 for ECL) was used, rated on an 8-point Likert scale.
- Perceived usefulness of understandability (PUU): Based on the Technology Acceptance Model (TAM) [36], PUU assesses the perceived usefulness and mental effort associated with different modularization types in process models. For RQ2, PUU was measured using an adapted questionnaire from [10], consisting of four items rated on an 8-point Likert scale.
- Perceived ease of use (PEU): PEU is based on TAM. It comprises the learnability as well as the usefulness of the modularization type of the process model. Less mental effort is involved when using a specific process model modularization type. To measure PEU, a questionnaire from [10] was used. PEU consists of four items. These items were investigated for RQ2 and evaluated by the participant using a 8 point Likert scale (ranging from totally agree (7) to totally disagree (0)).
- Intention to use (IU): The willingness to use a specific process model modularization type is indicated by the IU. The items of IU were taken from the questionnaire by [37] and adapted to the study context (i.e., process models). This serves the purpose that the questions are understandable and answerable for the participants. Two items for IU were queried. All items regarding comprehensibility were investigated for RQ2. They were evaluated by the participant using a 8 point Likert scale (ranging from totally agree (7) to totally disagree (0)).
- Solving duration: The time needed to complete a task. In this study, the first eye movement when performing a task was set as the starting point. The end point comprised the feedback in which the activity was found. The data were gathered via the recording methods (i.e., eye-tracking, audio) that were utilized for all four tasks of the process models (i.e., P1–P6). The measures of the solving duration were investigated for RQ3.
- Number of fixations: Fixations occur when the eyes remain relatively stationary on an object during information processing [38]. The number of fixations can provide insights into cognitive processes, such as attention focus, while reading objects like process model activities. A higher number of fixations suggests more cognitive processing is involved in comprehending the process model. The analysis of fixation numbers was part of RQ3.

To identify the comprehension strategies, data were collected using the following:

- Think-aloud: The think-aloud approach is instrumental in gaining insights into the strategies employed by participants while performing tasks. There are two primary types of think-aloud protocols: concurrent and retrospective [39,40]. The retrospective protocol is conducted post-task completion, where participants reflect and verbalize their thoughts after the task. This method can result in information loss or inaccuracies since participants may not recall all details accurately. On the other hand, the concurrent protocol happens in real-time as the task is being processed. This approach captures immediate thoughts and strategies, ensuring no loss of information. However, it can increase the cognitive load on participants as they have to articulate their thoughts while performing the task. Additionally, the act of reporting might influence their natural behavior [40].
  For this study, the concurrent protocol was chosen due to its ability to provide direct insights into the thought process. This choice is crucial because capturing each step in the comprehension process is vital. The protocol’s effectiveness also hinges on the instructions given for the think-aloud process. As noted by [41], there are two instruction types: spontaneous and instructed evaluation conditions. In the instructed evaluation, participants are periodically reminded to verbalize their thoughts, whereas in the spontaneous evaluation, such prompts are absent.
  In our study, participants were not reminded to verbalize during task performance as uninterrupted comprehension strategies were the focus. Reminders could disrupt the thought process, potentially leading to resumed viewing and reporting that does not accurately reflect the participant’s genuine comprehension strategy. This could result in the emergence of artificial strategies or misinterpretation of the cognitive process.
  Additionally, the study utilized a simplified instruction set for the think-aloud process. Participants were encouraged to articulate anything that came to mind during the task. This approach was integral to investigating Research Question 4 (RQ4).
  Another key measure in this study was the scan path, which refers to the sequence in which process model elements are fixated by the participants. It is essentially a series of fixations and saccades (eye movements). Understanding the scan path is crucial for RQ4 as it reveals the order in which information is processed and can provide deeper insights into the cognitive strategies employed by the participants.

**Figure 6:**
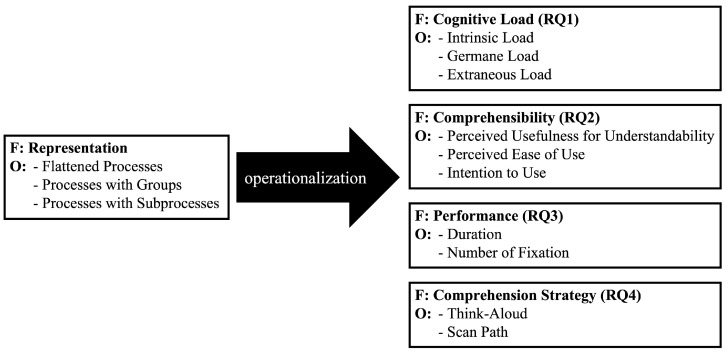
Research model (with F = theoretical factor and O = operationalization of factor).

Figure 6 summarizes all performance measures in a research model.

### 4.5. Instrumentation

In the study, different instrumentations (e.g., microphones) were utilized. As handwritten signatures were obtained, declarations (i.e., COVID-19 declaration) were applied paper-based. In contrast, textual descriptions (e.g., introductions), such as questionnaires, were provided online on a tablet (i.e., iPad Pro 12.9”) in LimeSurvey [42]. Next to instrumentations with direct participant interactions (i.e., through manual input), further instrumentations were applied. First, sound recordings were generated by a microphone built into the notebook (i.e., MacBook Pro 13.3”). Second, the slides including the process models were visualized on a DELL UltraSharp 49” monitor (i.e., resolution 5120 × 1440, Hz = 60). Third, eye-tracking data were collected by a combination of hard- (i.e., eye-tracking headset) and software (i.e., data collection, analysis, and visualization) from Pupil Labs [43]. The eye-tracker was a Pupil Core eye-tracking headset with a 120 Hz eye camera. For data collection (i.e., recording, calibration), Pupil Capture was utilized. Regarding the first data analysis and visualization of the eye-tracking recordings, Pupil Player was used. Fourth, sound and eye-tracking recordings were superimposed for further analysis in the application Shotcut [44]. Finally, statistical analysis of the recordings such as the data obtained by the questionnaires was performed using SPSS 27.

### 4.6. Study Design

Prior to the study, a pilot study was conducted in order to ensure the quality, comprehensibility, and feasibility of the study. The materials and methods were in compliance with the guidelines of the Ethics Committee of Ulm University (No. 424/21).

During the study, each session was conducted with only one participant at a time to ensure individualized attention and data accuracy. Participants were systematically categorized into one of six distinct groups. These groups were determined based on the sequence in which the participants engaged with the different modularization types (flattened process models ➀, process models with groups ➁, and process models with subprocesses ➂). This grouping strategy was part of a within-subject design, allowing each participant to experience all types of modularization in a predefined order.

Given that the study took place amidst the COVID-19 pandemic, strict adherence to the prevailing health and safety regulations was a top priority. These measures were implemented to safeguard the health of both the participants and the researchers involved.

**Figure 7:**
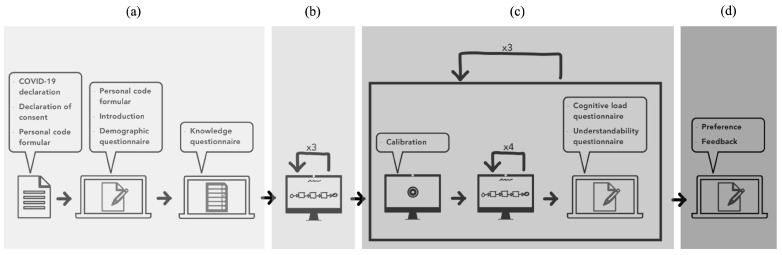
Applied study design: (**a**) general data and information, (**b**) task introduction, (**c**) task performance, and (**d**) final data.

On average, each study session lasted about 45 min. The structure of these sessions was meticulously planned and executed as depicted in Figure 7. This figure outlines the key segments of the study, which included general data and information collection, task introduction, actual task performance, and final data collection. This systematic approach ensured that each session was conducted efficiently and consistently, providing valuable and reliable data for analysis. Following, the procedure is presented based on the following segments:

1. General data and information: At the beginning of each study session, general data and information were collected (see Figure 7a). First, the participant had to sign a COVID-19 declaration. Afterwards, the declaration of consent was signed by the participant. The participant was instructed about (e.g., voluntariness, risks of study) study-specific topics (e.g., think-aloud approach, further study structure). Then, the demographic data of the participant (e.g., gender, age, prior knowledge) were collected. If the participant had prior knowledge in process modeling, a knowledge test had to be accomplished (see Section 4.2).
2. Task introduction: According to Figure 7b, the participant received an introduction regarding the task (i.e., search for activities in the process model while using the think-aloud approach) like “Find the activity ‘Pay purchase’”. To prepare the study participant for the subsequent tasks, a trial run with three objectives was carried out. Each objective was performed once and in the following modularization type order: ➀, ➁, and ➂. After completing an objective, the study participant was instructed on how to use the think-aloud approach correctly if it had not been used correctly beforehand.
3. Task performance: Subsequently to the task introduction, the study was carried out. This procedure is described in the section task performance in Figure 7c. In total, six process models were shown consecutive according to the clusters in Table 2 in Section 4.3 (i.e., two for each modularization type). The order in which the modularization types were shown depended on the group the participant was assigned to. The following procedure was performed six times: after a successful calibration, the recording by the eye-tracker and the recordings of the voice began. During recording, the participant searched for four activities in a process model (e.g., like in task 4 in P1 for the activity “Prepare food”) according to the defined order of the process models P1–P6. While searching for activities, the participant verbalized their strategy for the think-aloud approach (e.g., like “I think the activity ‘Prepare food’ is at the beginning of the process model”). The search of an activity was considered complete by the message that the activity was found through the participant (e.g., like “I found the activity ‘Prepare food’”). After completing the search of all four activities in a process model, the recordings were stopped. Then, the participant had to answer questionnaires regarding confronted cognitive load and process model comprehensibility.
4. Final data: The modularization types were rated (i.e., liked less (1)–liked most (3)) according to the participants preferences (see Figure 7d). With regard to the rating, a value could only be assigned once. Further, the participant was able to provide feedback.

Figure 7 outlines the study design.

## 5. Results

This section presents the descriptive as well as inferential statistics of the results obtained from the study. To evaluate whether the differences seen in the descriptive results reach statistical significance, analyses of variances (ANOVAs) were performed. Moreover, the Greenhouse–Geisser correction (i.e., ξ<0.75) or Hynh–Feldt correction (i.e., ξ>0.75) were applied (i.e., the significant Mauchly’s sphericity test).

### 5.1. Descriptive Statistics

**Figure 8:**
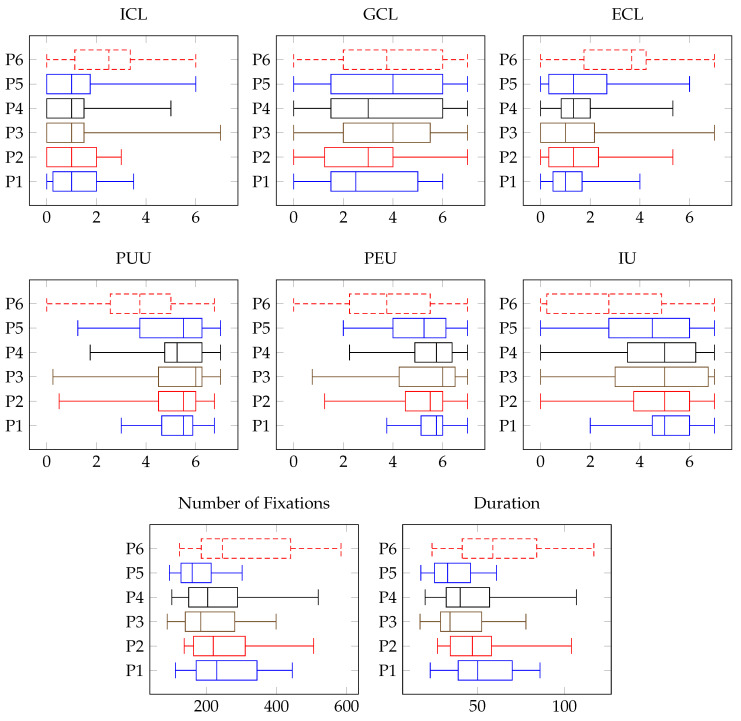
Comparison of process models (P1–P6).

Table 3 presents a detailed comparison of the mean (M) and standard deviation (SD) across various metrics: cognitive load (ICL, GCL, and ECL), comprehensibility (PUU, PEU, and IU), and performance (number of fixations, solving duration) for the process models (P1–P6). Additionally, the differences in these performance measures, such as ICL, are visually represented through box plot diagrams in Figure 8.

In terms of cognitive load, the results ranged from low (1.06 out of 8) to medium (3.92 out of 8), with GCL typically scoring higher than both ICL and ECL. As for comprehensibility, the study found minimal variance between the different process models. Most models maintained a medium level of comprehensibility (around 5.05 out of 8), except for P6, which was notably lower at 3.33 out of 8, indicating that it was perceived as more challenging compared to the others (P1–P5).

Table 4 further breaks down these findings by comparing the different modularization types. Here again, cognitive load scores varied from low (1.28 out of 8) to medium (3.63 out of 8). Comprehensibility consistently hovered around the medium level (4.80 out of 8). This outcome suggests that participants did not significantly differentiate between the benefits of modularization in their assessments or did not explicitly factor them into their evaluations.

### 5.2. Inferential Statistics

In the analysis, the within-subject factor “process model” (six levels: performance measures of RQ1–RQ3 for P1–P6) and the between-subject factor “modularization type” (three levels: flattened process models ➀, process models with groups ➁, process models with subprocesses ➂) were examined. Since not all process models (P1–P6) in the study had the same number for a modularization type, the little MCAR test was used as part of the data analysis. Based on the resulting data set, the repeated measure ANOVA was used for data analysis. Table 5 presents the performance measure results with respect to RQ1–RQ3 for P1–P6. The main effect (ME) of performance measures for P1–P6 (ME1) and for the modularization type comparison (ME2) were evaluated, along with the interaction effect process model ∗ modularization type (IE). In addition, in the event of significance for ME1, repeated contrasts were employed. Moreover, in the event of significance for ME2, post hoc analyses using the Bonferroni post hoc criterion were employed. Finally, all statistical tests were performed two-tailed, and the significance value was set to *p* < 0.05. The data for the individual process models and the data for the respective modularization types are based on the original dataset.

#### 5.2.1. Comparison of Process Models

In Table 5, ME1 presents the main effect for process models P1–P6 in terms of performance measures, which include Intrinsic Cognitive Load (ICL), Germane Cognitive Load (GCL), Extraneous Cognitive Load (ECL), Perceived Usefulness of Understandability (PUU), Perceived Ease of Use (PEU), Intention to Use (IU), number of fixations, and solving duration. Additionally, Table 6 gives an overview of the performance measures of the process models that demonstrated significant results concerning the main effects.

For ICL, a significant main effect was found (*p* < 0.003). Specifically, P1’s ICL was significantly lower compared to P6. Similarly, P2, P4, and P5 also reported significantly lower ICL than P6. In the case of ECL, a notable main effect was observed (*p* < 0.001), with P1, P2, P3, P4, and P5 having significantly lower ECL compared to P6.

Regarding PUU, a substantial main effect was recorded (*p* < 0.001). Here, P1, P2, P3, P4, and P5 scored significantly higher than P6. The trend continued with PEU, where a significant main effect (*p* < 0.001) was reported, and P1, P2, P3, P4, and P5 were rated significantly higher than P6. The same was true for IU, which exhibited a significant main effect (*p* < 0.001), with higher scores for P1, P2, P3, P4, and P5 compared to P6.

In terms of the number of fixations, a main effect was detected (*p* = 0.030). Here, P5 had a lower number of fixations than P1, P2, P3, and P6. Lastly, for solving duration, there was a main effect (*p* = 0.005), with P5 exhibiting a shorter solving duration than P1, P2, P3, and P6.

These findings highlight the varying impacts of different process models on cognitive load, comprehensibility, and performance measures, offering critical insights into the design and efficacy of process models.

#### 5.2.2. Consideration of Research Question RQ1–RQ3

This subsection presents the results with respect to RQ1–RQ3.

1. Results for RQ1
  For RQ1, all three dimensions of the cognitive load theory (i.e., ICL, GCL, and ECL) were analyzed. The Huynh–Feldt correction was utilized (i.e., significant Mauchly’s sphericity test) for comparisons over all process models with each other as ξ was higher than 0.75.
  (a) Regarding ICL, no significant main effect can be reported while comparing the modularization types with each other over all process models. For the comparison of the single process models, a difference in the ICL can be reported for P1 (*p* = 0.014). ➀ (M = 0.72, SD = 0.83) and ➁ (M = 0.89, SD = 0.78) had a lower ICL than ➂ (M = 2.07, SD = 1.06). In total, as no significant main effect was shown over all process models for ICL, the null hypothesis (i.e., $H_{0}:$ ➀ = ➁ = ➂) occurred. But, when comparing the ICL regarding the modularization types of the single process models, for P1, a significant difference was reported. Hence, the alternative hypotheses (i.e., $H_{1a-P1}:$ ➀ < ➁ < ➂) can be stated for P1.
  (b) Regarding GCL, no significant main effect can be reported for the comparisons over all process models. For the comparison of the single process models, no difference can be reported. In total, as no significant main effect was shown for GCL, the null hypothesis (i.e., $H_{0}:$ ➀ = ➁ = ➂) occurred.
  (c) Regarding ECL, no significant main effect can be reported while comparing the modularization types with each other over all process models. For the comparison of the single process models, no difference can be reported. In total, as no significant main effect was shown for ECL, the null hypothesis (i.e., $H_{0}:$ ➀ = ➁ = ➂) occurred.
2. Results for RQ2
  For RQ2, PUU, PEU, and IU were considered.
  (a) Regarding PUU, no significant main effect can be reported while comparing the modularization types with one another over all process models. For the comparison of the single process models, a difference in the PUU can be reported for P5 (*p* = 0.016). ➁ (M = 5.91, SD = 0.94) had a higher PUU than ➂ (M = 3.93, SD = 1.65). In total, as no significant main effect was shown over all process models for PUU, the null hypothesis (i.e., $H_{0}:$ ➀ = ➁ = ➂) occurred. But, when comparing the PUU regarding the modularization types of the single process models, for P5, a significant difference was reported. Hence, the alternative hypotheses (i.e., $H_{2a-P5}:$ ➂ < ➀ < ➁) can be stated for P1.
  (b) Regarding PEU, no significant main effect is given while comparing the modularization types with each other over all process models. For the comparison of the single process models, no difference can be reported. In total, as no significant main effect was shown for PEU, the null hypothesis (i.e., $H_{0}:$ ➀ = ➁ = ➂) occurred.
  (c) Regarding IU, no significant main effect is given while comparing the modularization types with each other over all process models. For the comparison of the single process models, a difference in the IU can be reported for P5 (*p* = 0.044). ➁ (M = 5.50, SD = 1.51) had a higher IU than ➂ (M = 3.15, SD = 2.14). In total, as no significant main effect was shown over all process models for IU, the null hypothesis (i.e., $H_{0}:$ ➀ = ➁ = ➂) occurred. But, when comparing the IU regarding the modularization types of the single process models, for P5, a significant difference was reported. Hence, the alternative hypotheses (i.e., $H_{2c-P5}:$ ➂ < ➀ < ➁) can be stated for P5.
3. Results for RQ3
  For RQ3, the number of fixations such as the duration while solving the four tasks for each process model (i.e., P1–P6) were considered.
  (a) Regarding the number of fixations, the clusters (see Table 2 in Section 4.3) were mapped in terms of the different modularization types (i.e., ➀, ➁, and ➂). Regarding the number of fixations, no main effect can be reported. No difference can be reported for the comparison between the modularization types. For the comparison of the single process models, a difference in terms of the number of fixations can be reported for P4 (*p* = 0.035) and P6 (*p* = 0.044). While comparing the process model modularization types, no significant difference was reported. In total, as no significant main effect was shown over all process models for the number of fixations, the null hypothesis (i.e., $H_{0}:$ ➀ = ➁ = ➂) occurred.
  (b) Regarding the solving duration, no significant main effect can be reported for the modularization types over all process models. Differences can be reported while comparing single process model modularization types. A difference is given for P4 (*p* = 0.041). The solving duration was higher for ➂ (M = 59.49, SD = 24.63) than for ➁ (M = 37.16, SD = 14.55) and ➀ (M = 37.62, SD = 13.49). A further difference can be reported for P6 (*p* = 0.034). ➀ (M = 40.09, SD = 17.26) was lower than ➂ (M = 72.49, SD = 25.55). In total, as no significant main effect was shown over all process models for the solving duration, the null hypothesis (i.e., $H_{0}:$ ➀ = ➁ = ➂) occurred. But, when comparing the IU regarding the modularization types of the single process models, for P4 and P6, a significant difference was reported. Hence, the alternative hypotheses (i.e., $H_{3b-P5}:$ ➀ < ➁ < ➂) can be stated for P5. Furthermore, the alternative hypothesis (i.e., $H_{3b-P6}:$ ➀ < ➁ < ➂) was given for P6.

In summary, when evaluating process model comprehension (addressed in RQ1 and RQ2) and performance (covered in RQ3), no significant main effect emerged from the comparison of different modularization types (➀, ➁, ➂). However, a closer examination of individual process models in terms of comprehensibility reveals supportive evidence for the use of modularization, particularly through the implementation of groups. This finding suggests that while the overall modularization type might not significantly impact comprehension and performance, specific approaches within modularization, such as grouping, can enhance the comprehensibility of process models.

In addition to the research questions RQ1–RQ3, the preference of the participants was of interest in terms of the modularization types. No significant differences can be reported regarding the preferences, F(2.00, 48.00) = 1.35, *p* = 0.270, and *η* = 0.053. Concerning the results, the following average values can be reported for the modularization types: M = 1.96 for ➀, M = 2.28 for ➁, and M = 1.84 for ➂. These results suggest that while there are variations in preferences for different modularization types, these differences are not statistically significant. This implies that participant preferences for one type of modularization over another are relatively balanced and do not strongly favor a particular style.

### 5.3. Comprehension Strategies

**Figure 9:**
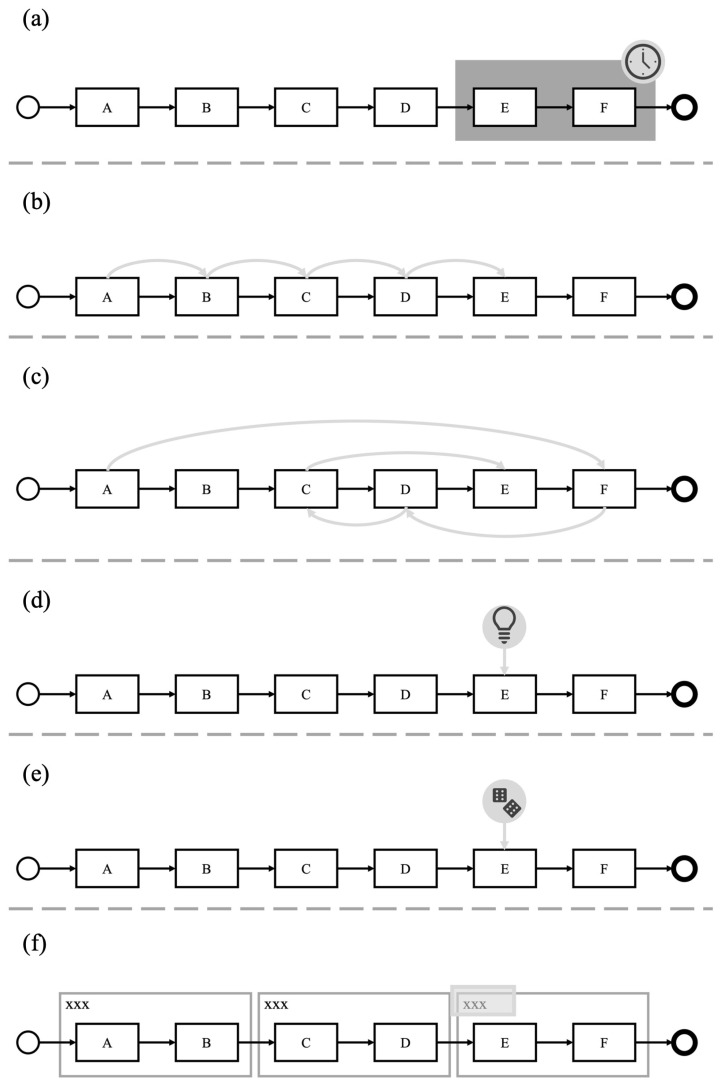
Comprehension strategies: (**a**) temporal allocation, (**b**) sequential execution, (**c**) erratic execution, (**d**) call to mind, (**e**) coincidence, and (**f**) thematic allocation.

In this section, we discuss the findings related to Research Question 4 (RQ4), which involved an in-depth analysis of the data gathered from eye-tracking and audio recordings. These recordings were integrated into videos, providing a comprehensive view of participant behavior during the tasks. Each video, corresponding to a specific task within each process model, was meticulously analyzed. The focus for eye-tracking data was primarily on the sequence of eye movements. This involved documenting the order in which participants viewed different activities in the process model, ascertained through both visual eye-tracking data and corroborated by audio recordings. For instance, if a participant first looked at one activity and then immediately moved to the adjacent activity, this sequence was noted. Audio recordings were scrutinized for specific keywords that participants used while navigating through the process models (refer to Table 7 for details). For example, “sequential” indicated a step-by-step approach through the process model. From this combined analysis of eye-tracking and audio data, various comprehension strategies employed by participants were identified. If multiple strategies were used for a single task, they were documented in the order of their occurrence. However, if the same strategy was employed consecutively for the same task, it was considered related. For example, in Figure 5a, if a participant first focused on activity A, then B, and subsequently C, this progression was noted as a continuous strategy. After the initial documentation, these strategies were reviewed and compared in detail. It was observed that some identified strategies were composites of different strategies and were thus removed from the analysis. Subsequently, all of the videos were re-examined to ensure that the remaining comprehension strategies accurately reflected the video content. This led to a second round of documentation for each task and participant. This rigorous analysis identified and outlined seven distinct comprehension strategies, offering valuable insights into how participants interact with and comprehend process models. In the following, the seven comprehension strategies are presented:

1. Temporal allocation
  The method for determining temporal allocation in the study involved participants associating activities with specific time segments within the process model, such as the beginning or end. This was achieved through a combination of verbal cues and eye-tracking data. Figure 9a illustrates an example of temporal allocation. Out of the total participants, 22 successfully engaged in this temporal allocation.
  This allocation process was primarily based on data obtained from the concurrent think-aloud approach, supplemented by eye movement analysis. Through the think-aloud method, participants often used keywords like “beginning” (referenced in Table 7) to describe their focus within the process model. When the quality of eye-tracking data was sufficient, these verbal indications were cross-referenced with the participants’ eye movements, particularly to verify if their gaze shifted to areas of the process model they verbally referenced, such as the start or end of the model.
  The eye movements, while beneficial for confirming the participants’ statements, were not always necessary for deducing temporal allocation as the verbal cues provided clear and direct indications of the participants’ focus areas within the process model. This approach allowed for a nuanced understanding of how participants navigated and conceptualized the process model in terms of time.
2. Sequential execution
  In the sequential execution strategy, participants systematically progress from one activity to the next adjacent one, maintaining a consistent direction throughout. This approach includes the possibility of traversing activities from left to right or vice versa. Figure 9b illustrates this method, labeled as erratic execution. For 23 participants, this sequential execution strategy was confirmed. This strategy was identified through a combination of data from the concurrent think-aloud approach and eye-tracking analysis.
  The concurrent think-aloud protocol played a crucial role in identifying this strategy, particularly through the use of specific keywords such as “sequential” (as referenced in Table 7). While some keywords were clear and unambiguous, others were more nuanced and required further interpretation. The determination of this strategy was strengthened by correlating these verbal cues with the participants’ eye movements, particularly observing their scan path as they moved from one activity directly to an adjacent one.
  In addition to specific keywords, the verbalization of each step in a sequential order by participants also aided in identifying this strategy. The combination of these verbal indicators with the eye movement patterns provided a comprehensive understanding of how participants engaged in sequential execution while navigating through the process model.
3. Erratic execution
  In the erratic execution strategy, participants engage with the activities in a non-sequential, unpredictable order. Instead of progressing to an adjacent activity after viewing one, they might choose to view activities that are spatially distant from each other. This approach allows for both left-to-right and right-to-left progression through the activities. Figure 9c visually represents this erratic execution. For 23 participants, the application of an erratic execution strategy was observed. This strategy was identified through a blend of data from the concurrent think-aloud approach and analysis of eye movements.
  The concurrent think-aloud approach was instrumental in this identification process, especially through the detection of specific keywords like “single activities”, as noted in Table 7. While some of these keywords were straightforward and clear, others presented ambiguity. The final determination of this strategy was made by marrying these verbal cues with the participants’ eye movements. Notably, the scan paths demonstrated movement from one activity to another non-adjacent activity.
  In addition to the keywords, participants’ verbal descriptions of engaging with single steps in a random, erratic order further solidified the identification of this strategy. This method provided a comprehensive understanding of how some participants approached the process model in a non-linear, erratic fashion.
4. Call to mind
  In the call to mind strategy, participants first familiarized themselves with the process model and then directly jumped to a specific activity or a cluster of activities that they remembered. This approach is illustrated in Figure 9d. Among the study participants, 15 were observed using this erratic execution strategy. The identification of this strategy primarily stemmed from the use of keywords such as “reminded”, as detailed in Table 7. This was discerned through the concurrent think-aloud approach.
  The verbal cues provided by the participants were clear and unequivocal, making the eye movement data a supplementary rather than essential component in identifying this strategy. However, when eye-tracking data was used, it showed that participants’ gaze moved directly to the activity or cluster of activities they recalled. This strategy highlights how participants leverage their memory of the process model to navigate efficiently, focusing on specific parts they remember rather than sequentially viewing each component.
5. Coincidence
  In the coincidence strategy, participants randomly come across the activity they are searching for. This strategy is depicted in Figure 9e. It was observed that 24 participants employed this approach, as proven by their actions during the study. The identification of the coincidence strategy was largely based on keywords such as “direct”, as listed in Table 7. This was ascertained through the concurrent think-aloud approach.
  While some keywords provided by participants were clear and straightforward, others were more ambiguous. The final determination of this strategy was made by integrating these verbal cues with the participants’ eye movement data. Notably, in this strategy, the participants’ gaze would fixate directly on the activity they were seeking without a prior systematic search.
  This approach indicates a more spontaneous and less structured method of navigating through the process model, where participants happen upon the required activity by chance rather than through a deliberate search pattern.
6. Creating overall picture
  In the creation of an overall picture strategy, participants aim to comprehend the entire process model comprehensively. This approach was observed in 21 participants. The strategy emerged from the analysis of data collected through the concurrent think-aloud approach, where participants articulated their desire to grasp the complete process.
  This strategy is characterized not just by a focus on individual activities but also on other elements such as roles within the process model. Participants using this approach often verbalized terms that indicated their intention to form a holistic comprehension of the process. A notable example of such a term is “overall picture”, as mentioned in Table 7. This keyword and similar expressions pointed to participants’ efforts to conceptualize the entire process model rather than concentrating on isolated parts or specific activities.
  This strategy reflects a more comprehensive and integrated approach to process model comprehension, where participants seek to synthesize all components of the model to form a cohesive comprehension of the entire process.
7. Thematic allocation
  In the thematic allocation strategy, participants categorize an activity within the context of a broader, generic topic. This approach is illustrated in Figure 9f. The strategy was confirmed in 23 participants. It was identified primarily through the data gathered via the concurrent think-aloud approach. During this process, participants used specific keywords like “allocate content”, as detailed in Table 7. These keywords were direct indicators of a thematic allocation approach, making them clear and unambiguous.
  As a result, the reliance on eye movement data was minimized in this case since the verbal cues provided sufficient information to ascertain the use of this comprehension strategy. However, when eye-tracking data was considered, it showed that participants’ gaze often focused on labels or titles, such as the designation of a collapsed subprocess or group, which aligns with the thematic allocation approach.
  One notable exception was observed in the thematic allocation context. A participant, while engaging with a flattened process model (➀), referred to a cluster during task resolution. Given that this modularization type does not typically include clusters, this suggests that the participant had mentally conceptualized a cluster to aid in their comprehension. This exception highlights the flexibility and diversity in how individuals process and interpret information in process models.

In addition to identifying various comprehension strategies, this study also analyzed how these strategies were utilized across different modularization types in reading process models P1–P6, as detailed in Table 8. The table shows the frequency of each comprehension strategy’s usage across all four tasks for each process model, broken down by modularization types (flattened process models ➀, process models with groups ➁, and process models with subprocesses ➂). For instance, in P1, the “temporal allocation” strategy was used four times for ➀, five times for ➁, and four times for ➂. The comparative analysis of usage frequency reveals that each comprehension strategy was employed across all modularization types. However, a notable variation was observed in the “thematic allocation” strategy, with significantly higher usage in ➁ (28 times) and ➂ (24 times), compared to just once in ➀. Additionally, strategies like “temporal allocation”, “call to mind”, and “creating overall picture” were not consistently used in every modularization type for all process models. For example, “temporal allocation” was not used in ➂ for P3. It is also important to note that participants often combined multiple comprehension strategies when searching for activities. These combined strategies ranged from one to five, with instances where the same strategy was used multiple times, interspersed with other strategies. This led to sequences of comprehension strategies, such as 67373 (“creating overall picture” (6), “thematic allocation” (7), “erratic execution” (3), “thematic allocation” (7), and “erratic execution” (3)), appearing multiple times. Table 9 outlines the direct predecessor (p) relationships between these strategies. For example, “temporal allocation” (1) often preceded strategies like “sequential execution” (2), “erratic execution” (3), “call to mind” (4), “coincidence” (5), and “thematic allocation” (6). However, “thematic allocation” did not precede “creating overall picture” (6), making combinations such as 12, 13, 14, 15, and 17 feasible. Interestingly, “creating overall picture” (6) was typically initiated at the start of a task, indicating that it rarely had any predecessor. This analysis of comprehension strategy usage and its sequences provides valuable insights into the cognitive processes and tactics employed by participants when interacting with different process models and modularization types.

### 5.4. Discussion

This study contributes to the existing body of research on process model comprehension by specifically focusing on how different types of modularization (flattened process models, process models with groups, and process models with subprocesses) affect comprehension. To explore this, four research questions (RQ1–RQ4) were formulated and investigated. These questions addressed the comprehensibility of process models both with and without modularization, encompassing ➀ (flattened process models), ➁ (process models with groups), and ➂ (process models with subprocesses).

In the analysis of individual process models (P1–P6), P6 emerged as notably more challenging to comprehend compared to the others. This was evident across several dimensions, including intrinsic and extraneous cognitive loads, perceived ease of use, perceived usefulness of understandability, intention to use, number of fixations, and solving duration, as detailed in Table 3 in Section 5.1. The think-aloud approach revealed that participants often verbally expressed their surprise or confusion about the placement of activities within P6. This phenomenon relates to the concept of expectation disconfirmation, which has been shown to influence behavior and experience in other research areas, such as web search [45].

A potential explanation for the difficulties encountered with P6 might lie in the participants’ prior knowledge or lack thereof. The scenarios presented in P1–P5 (such as refueling a car) are likely to be familiar and possibly part of participants’ daily experiences. In contrast, some participants may have been unfamiliar with the lending scenario in P6. But, prior knowledge plays a significant role in process model comprehension as it can greatly reduce cognitive load. This correlation between prior knowledge and ease of understanding is supported by previous studies [11,46]. Therefore, the increased difficulty in comprehending P6 could be attributed to a lack of prior knowledge about the lending process among the participants.

In RQ1, we evaluated the impact of different modularization types of process models (➀, ➁, ➂) on various aspects of cognitive load (CL)—intrinsic, germane, and extraneous. This assessment occurred after participants had engaged with each process model. When comparing these modularization types, the levels of intrinsic, germane, and extraneous cognitive load ranged from low to medium (as detailed in Table 4 in Section 5.1). This finding aligns with [14], which observed similar effects in simple process models with minimal complexity. As suggested by [47], process models varying in size and complexity could exhibit different cognitive load levels. Moreover, ref. [48] highlights the impact of information concealment in subprocesses, such as lowering abstraction levels and detailing activities within subprocesses. This can increase mental effort due to heightened abstraction and the split-attention effect. In our study, the intrinsic load reflected an average level of interactivity among process model elements. Participants’ prior knowledge about the content may have also influenced these results. Additionally, there were no apparent difficulties in forming mental models of the provided information, indicating a manageable germane cognitive load. This could be attributed to the size of the process models and the varying difficulties (e.g., content complexity, element interaction) they presented. The design of different modularization types was perceived as suitable, suggesting an appropriate level of extrinsic cognitive load. Particularly for ➂, an effective level of abstraction was employed. This is evidenced by the fact that differences in cognitive load were primarily noted in modularization types. For instance, in the subprocess model modularization (➂), the parent process was positioned similarly to ➀ and ➁, with subprocess models visually displayed below on the same page. A potential difference could arise from the concealment of subprocesses, as commonly seen in process modeling tools. Comparing modularization types revealed only one significant difference in cognitive load, specifically in the intrinsic cognitive load for P1. This discrepancy could be linked to element interactivity in ➂, which comprises multiple process models, possibly leading to the split-attention effect [49]. Since no other significant differences in cognitive load were observed, this study suggests that modularization types do not significantly impact cognitive load. This conclusion is consistent with findings from other studies (e.g., [8,10,14]), which propose that modularized process models may inherently possess intuitive comprehensibility.

In RQ2, the study focused on the comprehensibility and acceptability of various modularization types. This entailed examining Perceived Usefulness of Understandability (PUU), Perceived Ease of Use (PEU), and Intention to Use (IU). The findings, detailed in Table 4 in Section 5.1, indicated that both comprehensibility and intention to use were at a medium level overall. The consistently medium levels of PUU and PEU suggest that participants might have needed clarification about the comprehensibility of the different modularization types during their evaluation. This pattern aligns with the findings in [14], where PUU and PEU were also at medium levels for various modularization representations (horizontal, vertical, and orthogonal). Another possible explanation is that the process models had only minor variations in representation across different modularization types, with the differences mainly lying in a few elements, the style of modularization, and visualization. Specifically, for process model P5, the results for PUU indicated that ➁ (process models with groups) was more comprehensible than ➂ (process models with subprocesses). Although this was the only significant difference noted for PUU, participants generally rated ➁ as the most comprehensible and acceptable modularization type, followed by ➀ (flattened process models). This preference could be attributed to the fact that in ➁, all information is visualized in one location, thereby reducing the split-attention effect that is more likely to occur in ➀ and especially in ➂. In ➂, the split-attention effect arises because the information is distributed across different subprocess models, requiring participants to search through multiple process models. To maintain content completeness in ➂, additional elements (like start and end events and duplication of roles) are necessary, which might adversely affect comprehensibility. It is important to note that while ➁ enhances comprehensibility, it does not necessarily promote the reusability of individual modules as effectively as ➂. As for IU, no significant preference for a particular modularization type was observed, suggesting that process model readers do not have a strong inclination toward any specific type. However, the interaction effect (process model* modularization type) for IU revealed a tendency for participants to favor ➁ over ➂. This preference can be linked to the higher scores in PUU and PEU, indicating that participants tend to choose the most comprehensible approach.

In RQ3, we investigated the number of fixations and the time taken to solve tasks. The findings indicated a similarity between the performance metrics for ➀ (flattened process models) and ➁ (process models with groups). However, ➂ (process models with subprocesses) required a longer solving duration and a greater number of fixations. This could be attributed to the increased number of elements and the different visual arrangements of the process models, as discussed in RQ2. With more elements to consider in ➂, participants had more points to fixate on. Additionally, the use of the concurrent think-aloud approach meant that participants were verbalizing their thoughts and performance strategies while searching for activities, which naturally extended the time taken for task completion. The variation in how participants utilized the think-aloud approach could also contribute to differences in solving duration and fixation counts. These results align with the findings from RQ1 and RQ2, particularly regarding the preferences for modularization types.

In RQ4, seven distinct comprehension strategies were identified. These strategies could be employed either individually or in combination. Each strategy was used by more than half of the participants. Strategies like “temporal allocation”, “sequential execution”, “erratic execution”, “call to mind”, “coincidence”, and “creating overall picture” were frequently observed across all modularization types. While most comprehension strategies were applicable to all types of modularization, some differences were noted, particularly with “coincidence” in ➂. This modularization type enabled participants to find activities even without considering the parent process model, thereby bypassing a chronological sequence in subprocess models.

“Thematic allocation” was only used once in ➀. When applied to flattened process models, this strategy led to the creation of thematic clusters, potentially increasing cognitive load. Furthermore, the sequencing of comprehension strategies was analyzed. Most strategies followed a successor relationship, except for “creating overall picture”, where activities were found directly without the need for subsequent steps. Within the scope of this study, it remains unclear whether a series of several comprehension strategies offers any advantage over the use of a single strategy.

### 5.5. Limiting Factors

The following limiting factors were encountered during the study:

1. Limited process models
  Limitations regarding the process model complexity (i.e., number of activities, degree of difficulty) were given. The process models created were kept simple, and the number of process model elements (i.e., activities) was low. Hence, the participants completed the task in a short period of time and without necessarily comprehending the process models.
2. Process model representation
  The modularized process models were visualized on one level. For ➂ (process models with subprocesses), no information hiding was given. Therefore, there was no guarantee that the parent process model would be read chronologically before the subprocess models were read.
3. Differences in prior knowledge
  Even if participants have no prior knowledge, they comprehend the process models. But, according to studies from [50], novices need more completion time than experts. Further, if participants do not know the terms for process model elements, they may use a comprehension strategy that is not assigned to the appropriate method due to the words chosen.
4. Sample size
  Only N = 25 participants took part in the study. A higher sample size (i.e., number of participants) could lead to a different result. For example, further participants may have utilized additional comprehension strategies that had not yet been not identified. Next to the number of participants, the gender of the participants was unbalanced. N = 22 participants were male, whereas N = 3 participants were female. Hence, gender-specific differences could not be considered.
5. Usage of think-aloud approach
  The participants differed in terms of the level of detail in reporting the procedure. The usage of the concurrent think-aloud approach varied from low up to high depending on the participant. In addition, the reported content also differed. This could lead to discrepancies (e.g., in terms of times) as well as a possible lack of comprehension strategies.
6. Number of comprehension strategies
  It was not defined how the participant has to go through the process model while searching for an activity. Hence, participants often used more than one comprehension strategy. The impact of each comprehension strategy on performance cannot be traced back.
7. Data loss
  Data were lost due to technical problems. In some cases, the sound was not fully recorded (e.g., noise level of laptop cooling fan). Furthermore, occasional, as well as eye-tracking, images were not fully recorded (e.g., loose contact of the eye-tracker). Data loss was recognized during the data evaluation (i.e., watching and analyzing the recordings). An indicator for the loss of eye-tracking images was that fixations were not visualized. When data loss occurred, the eye-tracking data was not included in the evaluation of fixations. For a process model (P1–P6), the average data ***loss*** regarding sound records comprised 4.79 participants and regarding eye-tracking recordings 3.33 participants. Hence, based on the occurrence of data loss, incorrect conclusions and results may have resulted.

### 5.6. Future Work

The combined eye-tracking and concurrent think-aloud study has provided valuable insights into cognitive factors such as comprehensibility, cognitive load, and procedural approaches in the context of process model comprehension. There are several promising directions for future research:

Replicating the Study in a Practical Context: Applying the study in real-world settings would be beneficial. First, process models used in practice tend to be more complex than those created for this study. Second, employees in companies may have varying levels of knowledge about process models and their representations. Third, implementing the study with actual business process management tools instead of slide presentations could offer a more realistic scenario, especially in terms of information hiding and navigating different process levels.

Testing Single Comprehension Strategies in Scientific Institutions: Investigating individual comprehension strategies can provide insights into their advantages and limitations. For example, understanding when a strategy like “temporal allocation” is most effective, possibly in scenarios where prior knowledge is available, can be valuable. This exploration could also yield practical guidance on utilizing these strategies effectively.

Exploring Combinations of Comprehension Strategies: Researching how different comprehension strategies can be combined effectively is another intriguing area. This could lead to developing guidelines for the more efficient comprehension of process models.

Examining Different Modularization Approaches: Expanding the research to include various modularization approaches, such as horizontal modularization, may uncover additional comprehension strategies and combinations, enhancing our understanding of process model comprehension.

Considering Additional Process Model Notations: Investigating other process model notations, like Event-driven Process Chains (EPCs), which have different designs and modeling elements, could reveal new comprehension strategies tailored to these notations.

Improving the Think-Aloud Approach: Prior to the study, providing participants with a list of various elements and their standard terminology could standardize responses, leading to the more accurate mapping of comprehension strategies. The consistent use of terminology by participants would aid in more precise data interpretation and strategy identification. Overall, in terms of improving the think-aloud approach, implementing all of the tips from [20] (e.g., assurance of a warm-up training) into the methods and evaluation could have a positive impact in terms of capturing the cognitive processes.

These future research directions have the potential to deepen our understanding of how different individuals interact with and comprehend process models, contributing to more effective and user-friendly process model design and usage.

## 6. Conclusions

This paper investigates the impact of modularized BPMN 2.0 process models, including flattened process models, process models with groups, and process models with subprocesses, on various cognitive factors, such as comprehensibility, cognitive load, and procedural approaches. The study involved 25 participants in a combined eye-tracking and concurrent think-aloud study. Four research questions were explored, focusing on cognitive load, comprehensibility, performance, and comprehension strategies. The results revealed similar cognitive load, comprehensibility, and performance effects while reading process models. Process models with groups, notably flattened process models, emerged as the most comprehensible models and exhibited superior performance based on the study’s findings. An intriguing discovery was the identification of seven comprehension strategies employed by participants during the process model comprehension task. These comprehension strategies can be utilized individually or in combination, offering valuable insights into how individuals approach and comprehend process models.

## Figures and Tables

**Table 1:** Sample description.

Demographic Information	Number (n) of Participants	Details
Participants (at the location)	20	Ulm University
	5	University of Würzburg
Gender	22	male
	3	female
	0	divers
Age	2	<25
	21	25–35
	2	35–45
Graduation	9	bachelor’s degree
	14	master’s degree
	2	Ph.D.
Course studies	14	computer science
	3	economics
	2	economics and computer science
	2	design
	4	others
Prior knowledge	18	prior knowledge
	7	no prior knowledge
Average knowledge test result	18	6.05 out of 10 points

**Table 2:** Process scenario details.

Process Scenario (Process Number)	Number of Activities	Page Type	Sequence (Cluster)	Tasks
Flower purchase (-)	12	Trial	1 (-)	3
Refuel car (P1)	15	Task	2 (A)	4
Order food (P2)	10	Task	3 (A)	4
Prepare vacation (P3)	15	Task	4 (B)	4
Handling car breakdown (P4)	13	Task	5 (B)	4
Customer service coffee shop (P5)	10	Task	6 (C)	4
Lending (P6)	14	Task	7 (C)	4

**Table 3:** Comparison of process models (P1–P6).

Performance Measure	P1		P2		P3		P4		P5		P6		Sum P1–P6	
	M	SD	M	SD	M	SD	M	SD	M	SD	M	SD	M	SD
ICL *	1.15	1.05	1.06	0.95	1.35	1.63	1.15	1.21	1.29	1.50	2.40	1.59	8.40	7.93
GCL	3.08	1.93	3.00	1.66	3.88	2.22	3.77	2.31	3.92	2.33	3.75	2.24	21.40	12.69
ECL *	1.17	0.95	1.56	1.39	1.67	2.06	1.42	1.18	1.89	1.69	3.33	1.93	11.04	9.20
PUU *	5.20	0.96	5.08	1.39	5.07	1.84	5.39	1.29	4.89	1.58	3.51	1.92	29.14	8.98
PEU *	5.58	0.80	5.24	1.31	5.19	1.77	5.49	1.22	5.08	1.44	3.65	1.96	30.23	8.50
IU *	5.15	1.28	4.94	1.62	4.52	2.36	4.56	1.96	4.33	2.17	2.83	2.35	26.33	11.74
Eye ^a^ *	258.80	113.63	255.67	106.13	226.67	90.98	228.73	106.47	172.07	62.16	278.00	153.83	1419.94	633.20
Time ^b^ *	52.28	19.53	49.70	19.89	42.80	16.65	42.56	20.68	35.15	12.96	58.99	29.25	281.48	118.96

^a^ Eye = number of fixations. ^b^ Time = solving duration in (seconds.milliseconds). * = significance.

**Table 4:** Comparison of modularization types.

Performance Measure	Process		Group		Subprocess	
	M	SD	M	SD	M	SD
ICL	1.83	1.88	1.28	0.97	1.69	1.16
GCL	3.46	2.00	3.39	2.02	3.63	2.08
ECL	1.72	1.37	1.57	1.09	2.18	1.58
PUU	4.84	1.08	5.38	1.07	4.42	1.73
PEU	4.97	1.18	5.47	1.17	4.76	1.57
IU	4.49	1.77	4.80	1.58	4.07	2.19
Eye ^a^	445.53	181.89	452.80	182.87	521.60	199.46
Time ^b^	43.56	17.18	44.71	16.53	52.51	18.94

^a^ Eye = number of fixations. ^b^ Time = solving duration in (seconds.milliseconds).

**Table 5:** Inferential statistics of performance measure.

RQ	PerM ^a^	E ^b^	F	*p*	η
1	ICL	ME1 *	F(2.54; 63.56) = 4.36	=0.011	0.148
		ME2	F(2.00; 25.00) = 0.56	=0.583	0.042
		IE	F(5.09; 63.56) = 0.92	=0.475	0.069
	GCL	ME1	F(3.00; 74.96) = 1.78	=0.158	0.066
		ME2	F(2.00; 25.00) = 0.14	=0.870	0.011
		IE	F(6.00; 74.96) = 1.19	=0.321	0.087
	ECL	ME1 *	F(3.03; 75.72) = 6.84	<0.000	0.215
		ME2	F(2.00; 25.00) = 0.87	=0.433	0.065
		IE	F(6.06; 75.72) = 1.03	=0.415	0.076
2	PUU	ME1 *	F(2.86; 71.40) = 7.95	=0.001	0.208
		ME2	F(2.00; 25.00) = 1.60	=0.222	0.113
		IE	F(5.71; 71.40) = 0.89	=0.505	0.066
	PEU	ME1 *	F(2.95; 73.73) = 8.74	<0.000	0.241
		ME2	F(2.00; 25.00) = 1.13	=0.339	0.083
		IE	F(5.90; 73.73) = 1.08	=0.384	0.079
	IU	ME1 *	F(3.03; 75.70) = 6.20	=0.001	0.199
		ME2	F(2.00; 25.00) = 0.49	=0.618	0.038
		IE *	F(6.06; 75.70) = 2.32	=0.041	0.157
3	Fix ^c^	ME1	F(3.38; 84.47) = 8.29	<0.000	0.249
		ME2	F(2.00; 25.00) = 1.90	=0.170	0.132
		IE	F(6.76; 84.47) = 1.39	=0.224	0.100
	Dur ^d^	ME1 *	F(3.36; 84.01) = 8.35	<0.000	0.250
		ME2	F(2.00; 25.00) = 2.30	=0.121	0.156
		IE	F(6.72; 84.01) = 1.46	=0.195	0.105

^a^ PerM = performance measure. ^b^ E = effect (IE = ineraction effect; ME = main effect). ^c^ Fix = fixation. ^d^ Dur = duration. * = significance.

**Table 6:** Overview of process models (P1–P6) with significant results while comparing.

Performance Measure	Process Model			Compared Process Model			*p*
	PM	M	SD	PM	M	SD	
ICL	P1	1.15	1.05	P6	2.40	1.59	=0.001
	P2	1.06	0.95				=0.006
	P4	1.15	1.21				=0.015
	P5	1.29	1.50				=0.039
ECL	P1	1.17	0.95	P6	3.33	1.93	<0.001
	P2	1.56	1.39				=0.005
	P3	1.67	2.06				=0.021
	P4	1.42	1.18				=0.001
	P5	1.89	1.69				=0.033
PUU	P1	5.20	0.96	P6	3.51	1.92	=0.001
	P2	5.08	1.39				=0.008
	P3	5.07	1.84				=0.013
	P4	5.39	1.29				=0.001
	P5	4.89	1.58				<0.001
PEU	P1	5.58	0.80	P6	3.65	1.96	=0.001
	P2	5.24	1.31				=0.007
	P3	5.19	1.77				=0.008
	P4	5.49	1.22				=0.003
	P5	5.08	1.44				=0.003
IU	P1	5.15	1.28	P6	2.83	2.35	=0.002
	P2	4.94	1.62				=0.002
	P3	4.52	2.36				=0.024
	P4	4.56	1.96				=0.044
	P5	4.33	2.17				=0.003
Fix ^a^	P1	258.80	113.63	P5	172.07	62.16	=0.008
	P2	255.67	106.13				=0.036
	P3	226.67	90.98				=0.006
	P6	278.00	153.83				=0.028
Dur ^b^	P1	52.28	19.53	P5	35.15	12.96	=0.001
	P2	49.70	19.89				=0.020
	P3	42.80	16.65				=0.092
	P6	58.99	29.25				=0.006

^a^ Fix = fixation. ^b^ Dur = duration.

**Table 7:** Example of keywords in German (DE) and English (EN).

Comprehension Strategy	Keywords	Eye Movement (Optional)
Temporal allocation	DE: Anfang	
	EN: Beginning	
	DE: Vorne	
	EN: In the front	
	DE: Gegen Anfang	
	EN: Towards the beginning	
Sequential execution	DE: Sequentiell	
	EN: Sequential	
	DE: Schrittweise	
	EN: Step by step	
	DE: Von links nach rechts durchlaufen	Fixing activities in the chronological order
	EN: Go through from left to right	
Erratic execution	DE: Überspringen	
	EN: Skip over	
	DE: Einzelne Aktivitäten	Skip over single activities
	EN: Single activities	
	DE: Von links nach rechts durchlaufen	Skip over single activities
	EN: Go through from left to right	
Call to mind	DE: Erinnern	
	EN: Remind	
	DE: Schon gesehen	
	EN: Already seen	
	DE: War da	
	EN: Was there	
Coincidence	DE: Zufällig	Directly fixate searched activity
	EN: Coincidentally	
	DE: Direkt	
	EN: Direct	
	DE: Sogleich	
	EN: Instantly	
Creating overall picture	DE: Gesamtbild	
	EN: Overall picture	
	DE: Übersicht	
	EN: Overview	
	DE: Inhalt verstehen	
	EN: Understand content	
Thematic allocation	DE: Zugeordnet werden	
	EN: Be assigned	
	DE: Im ersten...	
	EN: In the first...	
	DE: Inhaltlich zuweisen	
	EN: Allocate content	

**Table 8:** Usage of the comprehension strategies.

Process Model	Comprehension Strategy						
	1 ^a^	2 ^b^	3 ^c^	4 ^d^	5 ^e^	6 ^f^	7 ^g^
P1	4; 5; 4	7; 3; 3	6; 5; 5	3; 3; 1	3; 3; 5	3; 0; 6	0; 1; 5
P2	7; 5; 5	5; 3; 2	5; 7; 6	2; 2; 0	5; 6; 5	3; 6; 5	0; 5; 2
P3	5; 4; 0	2; 2; 4	7; 4; 6	4; 1; 2	5; 5; 6	4; 4; 6	1; 6; 7
P4	3; 6; 4	3; 1; 1	5; 6; 7	3; 0; 0	3; 7; 4	4; 4, 6	0; 4; 3
P5	4; 5; 4	3; 4; 1	7; 5; 5	2; 2, 3	7; 6; 7	1; 4, 6	0; 3; 5
P6	4; 4; 4	2; 6; 1	7; 6; 5	0; 0; 0	4; 4; 5	1; 6; 4	0; 5; 2

^a^ 1 = Temporal allocation; ^b^ 2 = sequential execution; ^c^ 3 = erratic execution; ^d^ 4 = call to mind; ^e^ 5 = coincidence; ^f^ 6 = creating overall picture; and ^g^ 7 = thematic allocation.

**Table 9:** Combination of comprehension strategies (predecessor).

Comprehension Strategy	Predecessor Comprehension Strategy						
	1 ^a^	2 ^b^	3 ^c^	4 ^d^	5 ^e^	6 ^f^	7 ^g^
1 ^a^	/	p	p	p		p	
2 ^b^	p	/	p	p		p	p
3 ^c^	p	p	/	p	p	p	p
4 ^d^	p			/			
5 ^e^	p		p	p	/	p	p
6 ^f^						/	
7 ^g^	p	p	p	p		p	/

^a^ 1 = Temporal allocation; ^b^ 2 = sequential execution; ^c^ 3 = erratic execution; ^d^ 4 = call to mind; ^e^ 5 = coincidence; ^f^ 6 = creating overall picture; and ^g^ 7 = thematic allocation.

## Data Availability

The data presented in this study are available on request from the corresponding author, The data are not publicly available due to privacy restrictions.

## Appendix A

The following abbreviations are used in this manuscript:

➀	Flattened process models
➁	Process models with groups
➂	Process models with subprocesses
BPMN	Business process model and notation
CLT	Cognitive load theory
ECL	Extraneous cognitive load
E	Effect
Dur	Duration
Fix	Fixation
F	Theoretical factor
GCL	Germane cognitive load
ICL	Intrinsic cognitive load
IE	Interaction effect
IU	Intention to use
M	Mean
ME	Main effect
O	Operationalization of factor
P1	Process model 1
P2	Process model 2
P3	Process model 3
P4	Process model 4
P5	Process model 5
P6	Process model 6
PerM	Performance measure
PEU	Perceived ease of use
PUU	Perceived usefulness of comprehensibility
RQ	Research question
SD	Standard deviation
TAM	Technology Acceptance Model
